# Protein kinase Cepsilon is important for migration of neuroblastoma cells

**DOI:** 10.1186/1471-2407-8-365

**Published:** 2008-12-11

**Authors:** Helena Stensman, Christer Larsson

**Affiliations:** 1 Lund University, Center for Molecular Pathology, Dept of Laboratory Medicine, Malmö University Hospital, SE-205 02 Malmö, Sweden

## Abstract

**Background:**

Migration is important for the metastatic capacity and thus for the malignancy of cancer cells. There is limited knowledge on regulatory factors that promote the migration of neuroblastoma cells. This study investigates the hypothesis that protein kinase C (PKC) isoforms regulate neuroblastoma cell motility.

**Methods:**

PKC isoforms were downregulated with siRNA or modulated with activators and inhibitors. Migration was analyzed with scratch and transwell assays. Protein phosphorylation and expression levels were measured with Western blot.

**Results:**

Stimulation with 12-*O*-tetradecanoylphorbol-13-acetate (TPA) induced migration of SK-N-BE(2)C neuroblastoma cells. Treatment with the general protein kinase C (PKC) inhibitor GF109203X and the inhibitor of classical isoforms Gö6976 inhibited migration while an inhibitor of PKCβ isoforms did not have an effect. Downregulation of PKCε, but not of PKCα or PKCδ, with siRNA led to a suppression of both basal and TPA-stimulated migration. Experiments using PD98059 and LY294002, inhibitors of the Erk and phosphatidylinositol 3-kinase (PI3K) pathways, respectively, showed that PI3K is not necessary for TPA-induced migration. The Erk pathway might be involved in TPA-induced migration but not in migration driven by PKCε. TPA induced phosphorylation of the PKC substrate myristoylated alanine-rich C kinase substrate (MARCKS) which was suppressed by the PKC inhibitors. Treatment with siRNA oligonucleotides against different PKC isoforms before stimulation with TPA did not influence the phosphorylation of MARCKS.

**Conclusion:**

PKCε is important for migration of SK-N-BE(2)C neuroblastoma cells. Neither the Erk pathway nor MARCKS are critical downstream targets of PKCε but they may be involved in TPA-mediated migration.

## Background

Cell migration plays a central role in a wide range of different biological processes, both normal and pathological, including wound healing, inflammatory response and tumour metastasation [1]. The capacity of cells to migrate is dependent on signals from the extracellular environment which are transduced via networks of intracellular signal transduction proteins. A variety of intracellular signalling molecules including members of the protein kinase C (PKC) family of isoforms participate in the regulation of cellular migration [2-5].

PKC comprises a family of related serine/threonine kinases that are involved in a number of cellular processes such as proliferation and apoptosis in addition to their roles in regulating cellular morphology, adhesion and migration. Based on regulatory and structural properties, the PKC isoforms can be grouped in three different subfamilies; the classical PKCs (α, βI, βII and γ) are activated by Ca^2+^, phospholipids and diacylglycerol (DAG), the novel PKCs (δ, ε, η and θ) are activated by phospholipids and DAG but are insensitive to Ca^2+ ^while the atypical PKCs (ζ and ι/λ) require neither DAG nor Ca^2+ ^for activation [6].

An important role for PKC in cell migration has long been suggested for a wide range of cell types by the fact that phorbol esters, which are general PKC activators, enhance the motility of these cells [7-9]. Further studies have failed to pinpoint one or a few particular isoforms as being general regulators of migration [5]. It rather seems as if many isoforms have the capacity to influence the migratory behaviour and which isoform that is involved depends on the cell type. Overexpression of PKCα has been shown to increase motility in MCF-10 cells [10], 2C4 fibrosarcoma cells [11] and the breast cancer cell lines MCF-7 [12] and MDA-MB-435 [13] and PKCβI can mediate cytoskeletal rearrangements and platelet spreading on fibrinogen [14]. Activation of PKCδ with epidermal growth factor is important for migration of fibroblasts [15] and elevated levels of PKCδ contribute to a more metastatic phenotype of mammary tumour cells [16]. Finally, PKCε has been suggested to be important for glioma cell migration [17] and inhibition of PKCε leads to decreased motility of fibroblasts [18] and head and neck squamous cell carcinoma [19].

Neuroblastoma is the most common extracranial solid tumour among pediatric cancers affecting approximately 1 in 7000 live births [20]. The cancer is frequently lethal and this is coupled to widespread metastasation. It would therefore be beneficial to understand what regulates the migratory behaviour, which is one precondition for infiltration and spread, of neuroblastoma cells. This study was designed to investigate whether PKC isoforms can influence the migratory capacity of neuroblastoma cells and to elucidate putative pathways mediating the PKC effect.

## Methods

### Cell culture

Human SK-N-BE(2)C, KCN-69c and SH-SY5Y neuroblastoma cells were maintained in Minimal Essential Medium (Gibco) supplemented with 10% foetal bovine serum, 100 IU/ml penicillin and 100 μg/ml streptomycin (Gibco).

### Transfections with siRNA

Cells were transfected in 1 ml Optimem (Gibco) with 50 nM siRNA (Invitrogen) using 1.5 μl Lipofectamine 2000 (Invitrogen). The siRNA sequences are listed in Table 1. Transfections were interrupted after 6 h by adding 1 ml medium supplemented with 20% foetal bovine serum. The procedure was performed for three consecutive days after which optimal silencing was obtained as determined by Western blot analysis. Immunofluorescence studies have shown that the protein is downregulated to a similar extent in all cells in the culture (not shown).

**Table 1:** siRNA oligonucleotides

siRNA	Oligonucleotides
PKCα	CCGAGUGAAACUCACGGACUUCAAU
PKCδ	UUUCAAAGAGCUUCUCCAGGAUGUC
PKCε1	CACAAGUUCGGUAUCCACAACUACA
PKCε2	GCAAGGUCAUGUUGGCAGAACUCAA
PKCε3	CCACAAGUUCAUGGCCACCUAUCUU

### Migration assay

Cell migration was assayed in triplicates using a 48-well transwell setup (Neuroprobe) using polycarbonate Nucleopore filters with 8 μm pore size. The underside of the membrane was precoated with 20 μg/ml fibronectin (Sigma) in PBS for 16 h at 4°C. Cells were dissociated with trypsin (Gibco) for 5 min followed by addition of 0.1% soy bean trypsin inhibitor (Invitrogen). Cells were centrifuged, resuspended in serum-free medium and 15,000 cells were seeded in the upper chamber of each well. The lower chambers contained serum-free medium supplemented with activators or inhibitors at the following concentrations: 12-*O*-tetradecanoylphorbol-13-acetate (TPA; Sigma), 16 nM; GF109203X and Gö6976, 2 μM (both Calbiochem); LY333531, 200 nM (Alexis); PD98059, 50 μM and LY294002, 20 μM (both Sigma). Cells were incubated for 6 h in 37°C. Non-migrated cells on the upper side of the membrane were removed by scraping, while migrated cells attached to the underside of the membrane were fixed for 10 min in methanol and stained with Vectashield with DAPI (Vector laboratories). Cells were examined using a fluorescence microscope and all cells in a specified area in the middle of the membrane were counted.

### Scratch assay

Cells were seeded at a density of 450,000 cells per well in 12-well cell culture plates. After incubation for 24 hours, the confluent cell monolayer was scraped with a pipette tip creating a scratch in each well. Medium containing serum supplemented with TPA or inhibitors was added and cells were incubated at 37°C. For experiments with siRNA, 70,000 cells were seeded in 12-well cell culture plates and treated with siRNA as described and 18 hours after the last transfection, cell monolayers were scratched. Cells were photographed at different time points and the scratch area was measured using ImageJ.

### Western blot

1.0 × 10^6 ^cells were seeded in 60-mm cell culture dishes and incubated for 24 hours. Cells were pre-incubated for 1 h in serum-free medium prior to stimulation. Cells were washed twice in PBS and lysed in RIPA buffer (10 mM Tris-HCl, pH 7.2, 160 mM NaCl, 1% Triton X-100, 1% sodium deoxycholate, 0.1% sodium dodecyl sulfate, 1 mM EDTA, 1 mM EGTA) containing 40 μl/ml protease inhibitors (Roche Applied Science). Cells transfected with siRNA were lysed in the same way 18 h after the last transfection. Lysates were centrifuged for 10 min at 14,000 × g at 4°C. Proteins were electrophoretically separated on a 10% NuPAGE Novex Bis-Tris gel (Invitrogen) and transferred to a polyvinylidene diflouride membrane (Millipore). For detection, membranes were incubated with primary antibodies against phospho-MARCKS (1:500), phospho-Erk (1:500), Erk (1:500) (all Cell Signaling), MARCKS (1:1000) (Upstate), PKCα (1:3000), PKCβII (1:500), PKCδ (1:500) or PKCε (1:500) (all Santa Cruz Biotechnology) followed by incubation with a horseradish peroxidase-labelled secondary antibody (1:5000) (Amersham Biosciences). Horseradish peroxidase was thereafter visualised using the SuperSignal system (Pierce) as substrate. The chemoluminescence was detected with a CCD camera (Fujifilm).

### Calculations and statistics

IC_50 _values were calculated by doing a curve fit analysis to the equation *y *= *A*/(1+*x*/*B*) where *A *is the maximal effect and *B *is the IC_50 _value. Statistical analyses were done by doing ANOVA followed by Duncan's multiple range test using p < 0.05 as level of for significance.

## Results

### Activation of PKC stimulates migration of neuroblastoma cells

To investigate a putative role of PKC in neuroblastoma cell motility, the migration of SK-N-BE(2)C neuroblastoma cells was studied using transwell and scratch assays.

**Figure 1:**
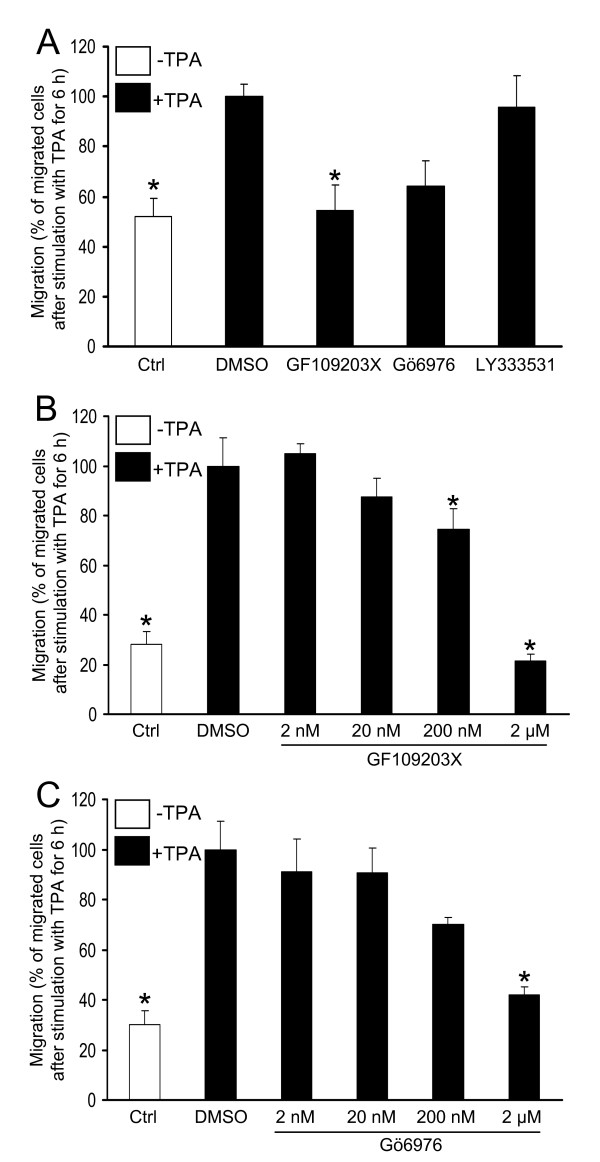
**Activation of PKC induces migration of SK-N-BE(2)C neuroblastoma cells**. (A) SK-N-BE(2)C cells seeded in serum-free medium in the upper wells of a migration chamber. were allowed to migrate for 6 h towards either serum-free medium without supplements (Ctrl) or towards serum-free medium with 16 nM TPA in the absence or presence of the PKC inhibitors GF109203X (2 μM), Gö6976 (2 μM) or LY333531 (200 nM). (B and C) SK-N-BE(2)C cells were allowed to migrate for 6 h towards 16 nM TPA in the presence of increasing concentrations of (B) GF109203X and (C) Gö6976. Data, the number of migrated cells expressed as percent of the number of migrated cells after TPA treatment in the absence of inhibitors, are mean ± SEM of four (A) or two (B and C) independent experiments. * denotes significant difference compared to TPA treatment in the absence of inhibitors.

SK-N-BE(2)C cells were seeded in the upper wells of the transwell assay and were allowed to migrate towards serum-free medium supplemented with 16 nM of the PKC activator TPA (Fig 1A). This is a treatment that does not lead to morphological changes of the cells [21,22] This demonstrated that TPA leads to a doubling of the number of migrated cells. Since TPA can influence other proteins than PKC isoforms [23], PKC inhibitors were included with TPA in the lower chamber to investigate if PKC activity mediates the TPA effect (Fig 1A–C). Both the general PKC inhibitor GF109203X and the inhibitor of the classical isoforms, Gö6976, markedly reduced the TPA-induced migration. The effects were concentration-dependent (Fig 1B–C) with 50% effect obtained with 310 nM for Gö6976 and 480 nM for GF109203X. The PKCβ inhibitor LY333531 did not influence the TPA effect at 200 nM.

**Figure 2:**
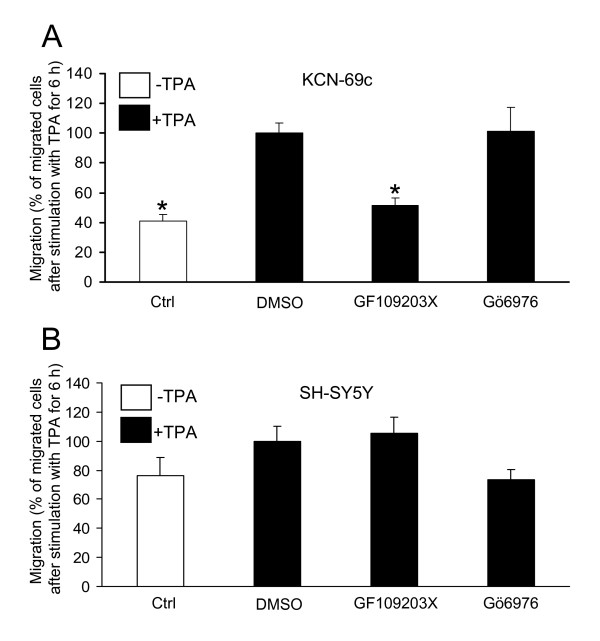
**Activation of PKC induces migration of KCN-69c cells but not of SH-SY5Y neuroblastoma cells**. KCN-69c (A) or SH-SY5Y (B) cells were seeded in serum-free medium in the upper wells of a migration chamber. The lower chambers were supplemented with DMSO (Ctrl), 16 nM TPA or 16 nM TPA with 2 μM GF109203X or 2 μM Gö6976 and cells were allowed to migrate for 6 h. Data, the number of migrated cells expressed as percent of the number of migrated cells after TPA treatment in the absence of inhibitors, are mean ± SEM of five (A) or four (B) independent experiments. * denotes significant difference compared to TPA treatment in the absence of inhibitors.

To analyse whether the PKC effect is general for neuroblastoma cells, we investigated migration in two other neuroblastoma cell lines, one *NMYC-*amplified (KCN-69c) and one without this amplification (SH-SY5Y) with the transwell assay (Fig 2). Addition of TPA led to increased migration of KCN-69c cells, an effect that was blocked by GF109203X whereas Gö6976 did not have an effect (Fig 2A). This indicates that a novel PKC isoform is important for migration of KCN-69c neuroblastoma cells. However, SH-SY5Y cells did not show a major migratory effect after activation of PKC (Fig 2B).

**Figure 3:**
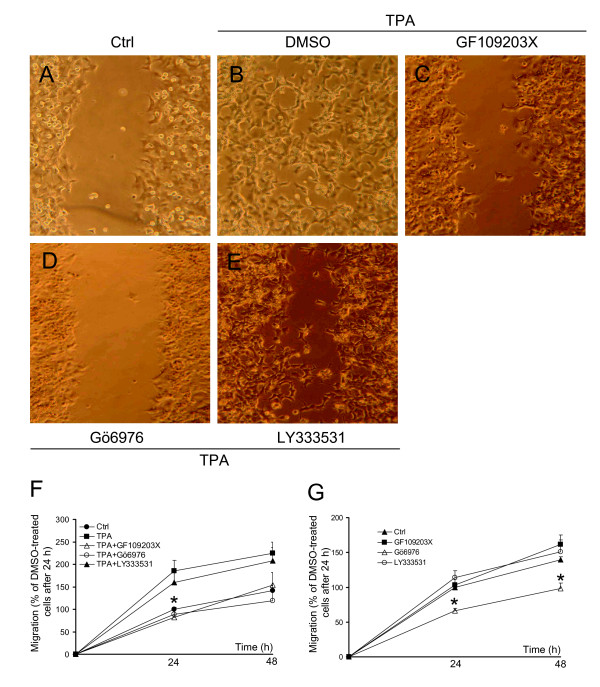
**Activation of PKC reduces the wound closure time of SK-N-BE(2)C neuroblastoma cells**. A confluent SK-N-BE(2)C cell monolayer was scraped with a pipette tip. (A-E) Cells were incubated in medium supplemented with serum (Ctrl) (A) or medium supplemented with serum and 16 nM TPA (B), or with the additional supplementation of PKC inhibitors GF109203X (2 μM) (C), Gö6976 (2 μM) (D) or LY333531 (200 nM) (E). Cells were incubated at 37°C for 48 hours and images of the scratches were captured. (F and G) Quantification of migration in the presence (F) or absence (G) of TPA. Data are presented as relative values where the 24 h timepoint for cells incubated in the absence of TPA is 100% and are mean ± SEM of four (F) or five (G) independent experiments. * denotes significant difference compared to absence of inhibitors.

To further establish the pro-migratory effect of PKC the cell motility was analysed with a scratch assay (Fig 3). Cells stimulated with TPA had almost completely closed the scratch after 48 hours (Fig 3B) contrasting the still visible scratch in cells incubated in the absence of TPA (Fig 3A). Both GF109203X and Gö6976 reduced the migration into the scratch (Fig 3C–D) demonstrating that the TPA effect is dependent on the activity of PKC. The PKCβ inhibitor LY333531 did not influence the TPA effect (Fig 3E). Quantitative analyses confirmed the observations (Fig 3F). Under basal conditions, *i.e. *in the absence of TPA, the inhibitor of classical PKC isoforms, Gö6976, reduced migration into the scratch while GF109203X and LY333531 were without effect (Fig 3G).

### PKCε is necessary for SK-N-BE(2)C cell migration

**Figure 4:**
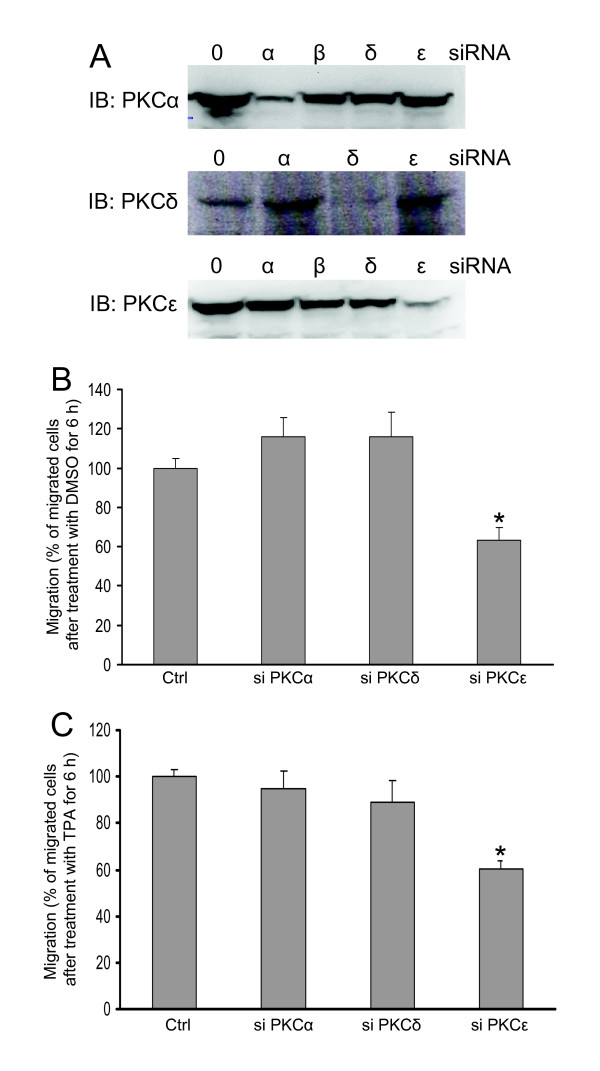
**PKCε mediates migration of SK-N-BE(2)C neuroblastoma cells**. (A) Cells were transfected with 50 nM siRNA oligonucleotides against PKCα, PKCδ and PKCε and the expression levels of the proteins were detected with Western blot. (B and C) SK-N-BE(2)C cells were treated with indicated siRNA and 18 hours after the last transfection 15,000 cells were seeded in the upper wells in a migration chamber. Cells were allowed to migrate towards serum-free medium (B) or 16 nM TPA (C) for 6 h. Data (mean ± SEM, n = 9 [B] or n = 11 [C]) represent the number of migrated cells expressed as the percent of migration obtained with cells that had been treated with no siRNA oligonucleotide. * denotes significant difference compared to control conditions.

To establish which isoform that mediates TPA-induced migration we used siRNA to reduce the levels of PKC isoforms. With this approach we could specifically reduce the protein levels of PKCα, PKCδ and PKCε (Fig 4A). However, despite trying four different siRNAs directed against PKCβ we were not able to reduce the expression of PKCβII in SK-N-BE(2)C cells (not shown).

SK-N-BE(2)C cells transfected with siRNAs were seeded in the upper wells of the transwell migration chambers and were allowed to migrate towards serum-free medium (Fig 4B) or medium supplemented with 16 nM TPA (Fig 4C). In both cases, treatment with the PKCε siRNA resulted in suppressed migration. Reduction of PKCα or PKCδ levels did not significantly influence migration.

**Figure 5:**
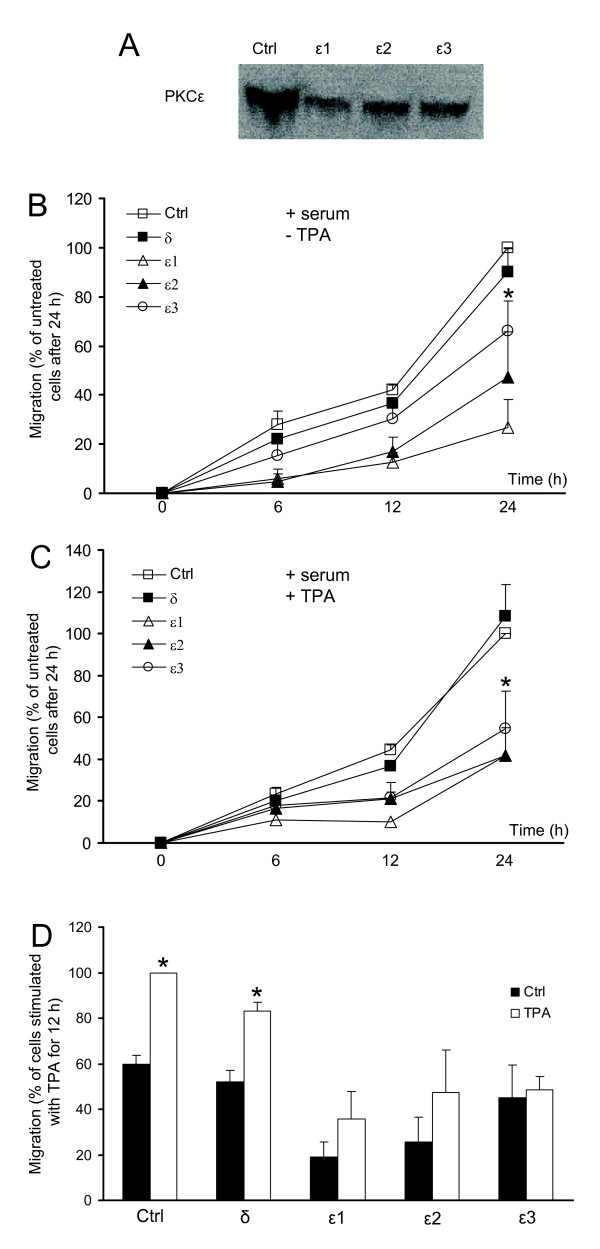
**PKCε is necessary for migration of SK-N-BE(2)C neuroblastoma cells**. (A) Cells were transfected with three different siRNA oligonucleotides against PKCε and the expression levels of PKCε were detected with Western blot. (B and C) Cells seeded in 35-mm cells culture wells were transfected with the PKCε siRNA oligonucleotides, one oligonucleotide against PKCδ or with an equal amount of water (Ctrl). Cells were incubated for 18 h after the last transfection and thereafter the confluent cell monolayer was scraped with a pipette tip. Medium with serum (B) or 16 nM TPA (C) was added to the wells. Data are presented as relative values of the migration of control cells after 24 h and are mean ± SEM of three independent experiments. (D) Data are presented as relative values where TPA at the 12 h time point is 100% and are mean ± SEM of three independent experiments. * denotes significant difference for all PKCε siRNAs compared to control conditions (B and C) and significant difference compared to absence of TPA (D).

To further confirm the role of PKCε we transfected cells with two other siRNA oligonucleotides against PKCε (ε2 and ε3), which both reduced the expression of PKCε (Fig 5A). A scratch assay with cells transfected with the different siRNA oligonucleotides against PKCε and with a PKCδ siRNA oligonuclotide as control was thereafter performed (Fig 5B–D). Cells were incubated with medium supplemented with serum alone (Fig 5B) or with serum and 16 nM TPA (Fig 5C). After 24 hours control cells and cells transfected with siRNA against PKCδ had migrated to the same extent. However, cells treated with either siRNA against PKCε had a reduced ability to close the scratch both in the absence and presence of TPA although the effects of the individual PKCε oligos differed somewhat (Fig 5D). These results clearly indicate that PKCε is necessary for migration of SK-N-BE(2)C neuroblastoma cells.

### Neither the PI3K pathway nor the Erk pathway is involved in PKCε-induced migration

**Figure 6:**
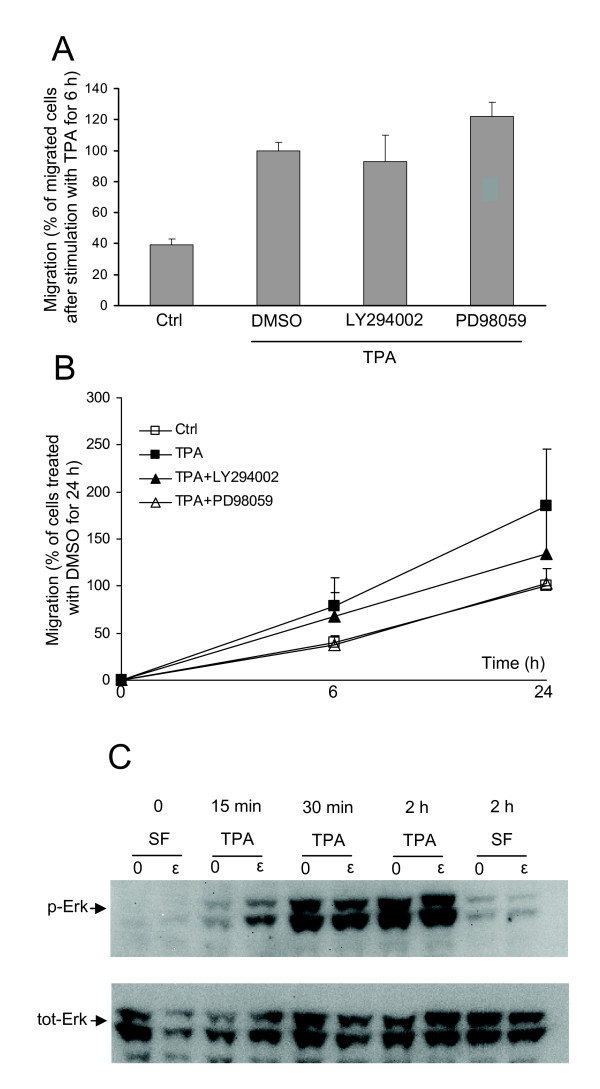
**Neither the Erk pathway nor the PI3K pathway is involved in PKCε-mediated migration of SK-N-BE(2)C cells**. (A) Cells were allowed to migrate for 6 h towards the lower chambers which were supplemented with DMSO (Ctrl) or 16 nM TPA in the absence or presence of the PI3K inhibitor LY294002 (20 μM) or the MEK inhibitor PD98059 (50 μM). Data, the number of migrated cells expressed as percent of the number of migrated cells after TPA treatment in the absence of inhibitors, are mean ± SEM of three independent experiments. (B) Confluent monolayers of SK-N-BE(2)C cells were scraped with a pipette tip. Cells were incubated in serum-containing medium (ctrl) and 16 nM TPA with or without 20 μM LY294002 or 50 μM PD98059. Data, migration into the scratch area, are presented as relative values where DMSO at the 24 h timepoint is 100% and are the mean ± SEM of three independent experiments. (C) Cells were transfected with 50 nM of siRNA oligonucleotide against PKCε (ε) or an equal amount of water (0) before stimulation with 16 nM TPA for different time periods. Thereafter cells were lysed and analysed by Western blotting using antibodies against phosphorylated Erk (p-Erk) and total Erk (tot-Erk).

The PI3K pathway and the Erk pathway have previously been shown to regulate the migration of neuroblastoma cells [24,25]. In particular PI3K is required for motility in many cell types suggesting a more universal importance of this signalling pathway for migration. It is therefore not unlikely that a basal activity of these pathways may be of importance for the migratory effect of TPA. To address this issue, we investigated whether activity in one or both of these pathways is important for the TPA-induced migration of SK-N-BE(2)C neuroblastoma cells using both transwell and scratch assays. Neither LY294002, a PI3K inhibitor, nor PD98059, an inhibitor of the Erk pathway, had an effect in the transwell assay (Fig 6A) whereas the there was a tendency towards reduced TPA-induced migration in the scratch assay in the presence of the MEK inhibitor (Fig 6B). The PI3K inhibitor had only a minor effect on migration into the scratch.

The fact that the PD98059 caused a tendency to reduced migration in the scratch assay led us to investigate whether Erk is a mediator of the pro-migratory effect of PKCε. However, TPA induced Erk phosphorylation to the same extent in control cells as in cells with downregulated PKCε (Fig 6C), indicating that Erk is not a crucial mediator of the PKCε effect.

### PKC-mediated phosphorylation of MARCKS

**Figure 7:**
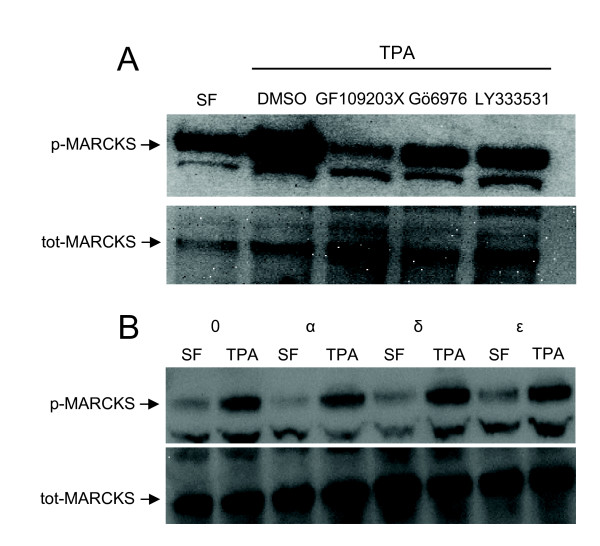
**Activation of PKC induces phosphorylation of MARCKS**. (A) SK-N-BE(2)C cells were incubated in serum-free medium for 1 h followed by pre-treatment with 2 μM GF109203X, 2 μM Gö6976 or 200 nM LY333531 for 5 min and thereafter stimulation with 16 nM TPA. Cells were lysed and analysed with Western blot using antibodies detecting phosphorylated (p-MARCKS) and total MARCKS (tot-MARCKS). (B) Cells were transfected for three consecutive days with 50 nM of siRNA oligonucleotides against PKCα, PKCδ and PKCε or an equal amount of water. 18 h after the last transfection cells were incubated with serum-free medium and thereafter stimulated with 16 nM TPA for 1 h. Cells were lysed and analysed by Western blotting using antibodies against phosphorylated and total MARCKS.

MARCKS is a PKC substrate which, depending on phosphorylation status, can bind F-actin and sequester phosphatidylinositol 4,5-bisphosphate and consequently regulate the cortical microfilaments [26]. To investigate whether MARCKS is phosphorylated during PKC-induced migration, SK-N-BE(2)C cells were treated with TPA and PKC inhibitors and the phosphorylation of MARCKS was analysed (Fig 7A). Stimulation with TPA for 1 h led to increased phosphorylation of MARCKS, which was suppressed by pre-treatment with PKC inhibitors (Fig 7A). Gö6976 and the PKCβ inhibitor LY333531 reduced MARCKS phosphorylation to levels seen in untreated cells and the general PKC inhibitor GF109203X suppressed them even further.

Cells were also transfected with siRNA oligos against PKCα, PKCδ and PKCε and stimulated with TPA for 1 h followed by analysis of MARCKS phosphorylation (Fig 7B). TPA treatment led to increased phosphorylation of MARCKS under all conditions indicating that several isoforms phosphorylate MARCKS in SK-N-BE(2)C cells.

## Discussion

A major problem in curing cancer is the capacity of cancer cells to migrate, invade tissues and subsequently seed metastases in other organs. This is also the case for neuroblastoma, a pediatric cancer derived from the peripheral sympathetic nervous system. The mechanisms determining the migratory capacity of neuroblastoma cells are not fully understood. Several reports indicate that growth factors, such as IGF-1 [27] and PDGF [25], and integrins [28] can stimulate neuroblastoma cell motility. In this study we demonstrate that a direct activation of PKC is sufficient to induce migration of neuroblastoma cells and PKC thus arises as an interesting target to suppress the motility of these cells.

Activation of PKC stimulated migration of two different neuroblastoma cell lines, SK-N-BE(2)C and KCN-69c, whereas the SH-SY5Y cell line did not increase its motility in response to PKC activators. This is not due to a poor migratory capacity of these cells since they migrate in response to other stimuli [25,27,28]. However, in terms of PKC effects SH-SY5Y cells are unique in that they differentiate upon treatment with TPA [29] which may explain why they do not migrate upon PKC activation. Another possible explanation is the fact that SK-N-BE(2)C and KCN-69c, but not SH-SY5Y cells, carry an *NMYC *amplification which results in more aggressive tumours [30]. The amplification may be associated with the presence of a pathway that transduces a PKC signal to increased motility. However, a larger panel of neuroblastoma cells is necessary to corroborate such a hypothesis.

PKC comprises a family of ten related isoforms, eight of which are TPA-sensitive, and of these, neuroblastoma cells generally express PKCα, PKCβII, PKCδ and PKCε [31]. Reducing the levels of PKCε, but not of PKCα or PKCδ, with siRNA inhibited migration both under basal conditions and when cells were stimulated with TPA. This is not due to off-target effects since three different siRNA oligonucleotides against PKCε all led to a reduced migration. Despite transfecting the cells with siRNA for three consecutive days we were not able to reduce the levels of PKCε completely which raises the possibility that even more suppressive effects could be obtained if PKCε could be depleted from the cells. A role of PKCε is in line with the suppression of the TPA effect obtained by the general PKC inhibitor GF109203X. However, in contrast to PKCε siRNA treatment, the kinase inhibitor did not affect migration under basal conditions. PKCε has been shown to induce morphological effects, induction of neurites [32] and dismantling of stress fibres [33], independently of its kinase activity. Our results indicate that also some of the promigratory effects of PKCε may be exerted independently of its catalytic activity.

The inhibitor of classical PKCs, Gö6976, also suppressed migration, indicating a potential role for these isoforms in migration. However, Gö6976 influenced migration both in the absence and presence of TPA contrasting the effect of GF109203X, which did not have an effect under basal conditions. Gö6976 has been shown to exert effects that are unrelated to and independent of PKC inhibition [34-36]. Furthermore, neither inhibition of PKCα with siRNA nor of PKCβ with LY333531 suppressed migration. This makes it more conceivable that PKCε is the primary promigratory PKC isoform in neuroblastoma cells and that Gö6976 inhibits motility by some other actions.

There are several different mechanisms through which PKCε may mediate its effects on cellular motility. Integrins are receptors for extracellular matrix components and are critically involved in the regulation of cell motility. PKCε has been shown to both regulate the recycling of integrins [18,37] and participate in down stream signalling following integrin clustering [17]. One of the putative PKCε targets is Erk which is targeted to focal adhesions following direct activation of PKC [38] or to focal complexes during HGF-mediated cell movement [39]. Both of these events are mediated via PKCε but our data do not support a critical role of Erk in PKCε-mediated migration of neuroblastoma cells. Although there was a tendency towards suppression of the wound healing by PD98059, it had no effect in the transwell assay and downregulation of PKCε to levels that cause a reduced migration did not influence TPA-stimulated Erk phosphorylation.

In addition to regulating other signalling proteins, PKC can also phosphorylate several proteins, such as MARCKS and ERM proteins [11,40], that more directly regulate the structure of the cytoskeleton. There was indeed a substantial PKC-mediated increase in MARCKS phosphorylation concomitant with TPA-stimulated migration indicating a role for MARCKS in the PKC-mediated motility of neuroblastoma cells. An involvement of MARCKS in PKC-regulated migration has been suggested in many other cell types [15,41,42] and our data would further support the general importance of this pathway.

However, experiments with siRNA showed that the phosphorylation of MARCKS was not altered when any of the isoforms PKCα, PKCδ or PKCε was downregulated. Since downregulation of PKCε leads to suppressed migration it does not seem as if MARCKS is specific and critical in the PKCε pathway. Instead it is conceivable that several isoforms phosphorylate MARCKS upon addition of TPA. This is further supported by the finding that the inhibitor of classical isoforms, Gö6976, partially reduces the phosphorylation whereas the general PKC inhibitor GF109203X has an even larger effect. MARCKS has been shown to be a high affinity substrate for both novel and classical PKC isoforms *in vitro *and in intact cells [43,44] supporting our finding that several PKC isoforms can phosphorylate MARCKS in SK-N-BE(2)C cells.

## Conclusion

In conclusion, we show for the first time that PKCε is necessary to promote migration of SK-N-BE(2)C neuroblastoma cells making it a possible target for blocking the motility of these cells.

## Competing interests

The authors declare that they have no competing interests.

## Authors' contributions

HS performed all experiments, participated in the design of the study and drafted the manuscript.

CL participated in the design of the study and drafting of the manuscript.

## Pre-publication history

The pre-publication history for this paper can be accessed here:


